# Redox Homeostasis in Poultry/Animal Production

**DOI:** 10.3390/antiox14111365

**Published:** 2025-11-17

**Authors:** Peter F. Surai, Anton Surai, Katie Earle-Payne

**Affiliations:** 1 Biochemistry Department, Vitagene and Health Research Centre, Bristol BS4 2RS, UK; 2 Faculty of Veterinary Medicine, Trakia University, 6000 Stara Zagora, Bulgaria; 3 Department of Animal Nutrition, Szent Istvan University, H-2103 Gödöllo, Hungary; 4 Physiology and Biochemistry Department, Saint-Petersburg State Academy of Veterinary Medicine, 196084 St. Petersburg, Russia; 5 Healthy Food Services, Bristol BS4 2NN, UK; 6 NHS Greater Glasgow and Clyde, Renfrewshire Health and Social Care Centre, 10 Ferry Road, Renfrew PA4 8RU, UK; 7 School of Pharmacy and Life Sciences, Robert Gordon University, Sir Ian Wood Building, Garthdee Road, Aberdeen AB10 7GJ, UK

Commercial animal/poultry production is associated with a range of stresses, including physiological, environmental, technological, nutritional, and internal/immunological stresses. It is practically impossible to avoid these stresses under the commercial conditions of poultry/animal production and the development of strategies for stress protection has become a hot topic in recent years [1]. Accumulating evidence indicates that, at the molecular level, most commercially relevant stresses in poultry and farm animals, including pigs and cows, are related to redox imbalances, compromised antioxidant defences, the overproduction of free radicals, and oxidative stress. Importantly, the poor reputation of reactive oxygen species (ROS) has been challenged and their involvement in redox signalling has become an important topic of current research and applications. It should be emphasised that stress adaptation is related to the activation of various transcription factors, including Nrf2 and NF-κB, and vitagenes [1]. In general, maintaining an optimal redox status is a key task for the integrated antioxidant defence network. In the stress-inducing conditions of commercial animal/poultry production, the internal antioxidant defence system is often incapable of mitgating the overproduction of ROS and needs external assistance, which can be provided by the dietary supplementation of traditional antioxidants such as vitamin E or other nutrients possessing relevant regulatory functions, including selenium, taurine, carnitine, polyphenolics, and others. An important task for nutritionists is to find an optimal balance of dietary antioxidants to provide animals with maximum antioxidant defences, effective stress signalling, and adaptation which are vital elements in gut health maintenance, immunocompetence, inflammation control, and the maintenance of a high productive and reproductive performance in animals/poultry [1].

This Special Issue is devoted to redox homeostasis maintenance in poultry/animal production to provide optimal health and high stress resistance/resilience under commercially relevant stress conditions. There were seven papers included in this Special Issue, including three reviews and four experimental papers.

An interesting paper devoted to the protective role of 25-Hydroxycholecalciferol (25-OH-D3) in obese chickens was published [2]. The dietary supplementation of 25-OH-D3 was reported to be associated with an improved redox status related to sustained Nrf2 activation and the maintenance of GSH concentrations, and decreased lipid (MDA) and protein (protein carbonylation) oxidation to potentiate cell survival in failing hearts [2]. Recently, the important antioxidant effects of 25-OH-D3 were demonstrated in broilers [3] and pigs [4,5,6]. Indeed, the involvement of various forms of vitamin D in redox balance maintenance, including the regulation of transcription factors (e. g. Nrf2 and NF-κB) deserves more attention [7,8].

The effects of oil quality and trace mineral source on the growth performance, antioxidant activity, and meat quality of growing–finishing pigs were investigated [9]. It was found that adding oxidised soy oil to the pigs’ diet detrimentally affected the ADG and dressing percentage of growing–finishing pigs. At the same time, replacing inorganic trace minerals (ITMs) with organic trace minerals (OTMs) and organic Se (selenium yeast) was proven to mitigate the aforementioned negative consequences of feeding oxidised oil to pigs by supporting antioxidant defences, as evidenced by increased serum CAT and GSH-Px activities in growing–finishing pigs [9]. Based on the recent review of the relative bioavailability of trace minerals in animal nutrition [10], it could be concluded that OTMs can be used in chicken diets at much lower levels than the current recommendations for ITMs without negatively impacting their performance and leading to a positive environmental effect due to the decreased excretion of excess minerals. However, the authors highlighted that not all OTMs are equal in terms of bioavailability and effectiveness [10], which also applies to pig nutrition [11]. In general, oxidised oils in poultry [12,13,14] and pig [15,16,17,18] diets are important nutritional stressors affecting the redox balance and antioxidant defences in the animal body and the search for nutritional supplements to address this problem continues. In paper [9], the inclusion of organic Se into the pigs’ diet formed a part of the protective nutrition. Interestingly, an organic Se study was described in another paper in this Issue. In particular, the protective effects of hydroxy-selenomethionine (OH-SeMet), selenium-enriched yeast (SeY), and sodium selenite (SeNa) against heat stress during reproductive cycle of sows were studied [19]. Compared with SeNa or SeY, OH-SeMet more effectively mitigated the adverse effects induced by heat stress in sows and their offspring. This led to the improved maintenance of offspring growth performance, as indicated by an improved number of live-born piglets, increased litter weight at day 21, and enhanced litter body weight gain from days 1 to 21. Similarly, in earlier publications, similar effects of organic Se were demonstrated: maternal OH-SeMet supplementation was indicated to increase the number of total born piglets, shorten the duration of farrowing, improve the antioxidant defences of sows and their offspring, as well as improve the growth performance of suckling pigs during the first week of lactation [20,21]. An important advantage of the paper by Wang et al. [19] is related to deep biochemical studies that explain the beneficial effects of organic Se. In particular, OH-SeMet was effective at supporting endogenous redox systems, as evidenced by enhanced levels of TXNRD and GSH and reduced levels of GSSG in the serum of sows, and increased T-AOC, TXNRD, and GSH simultaneously with reduced lipid peroxidation (MDA) and GSSG in the serum of piglets. Of note, OH-SeMet also increased T-AOC in the jejunum of piglets. The anti-inflammatory properties of OH-SeMet deserve special attention. In fact, OH-SeMet was more effective than SeY in regulating immune responses and reducing inflammatory markers (IL-1β and Il-6 in sows at d14 of lactation and in piglets at d21) in the serum of sows in comparison to SeNa [19]. Importantly, previous observations also demonstrated the anti-inflammatory properties of OH-SeMet in broilers under heat stress [22] and in macrophages in vitro [23]. In general, OH-SeMet was found to have protective effects against heat stress-induced barrier disruption and the inflammatory response in the jejunum of growing pigs [24]. In comparison to sodium selenite, OH-SeMet showed better gut protection for the sows’ offspring, as evidenced by a decreased crypt depth and enhanced villus height/crypt depth ratio in the duodenum, jejunum, and ileum. This was associated with an upregulation of the expression of selenoproteins (GPX6, TXNRD3, GPX4, and SELENON) and the tight junction protein (ZO-1) and the downregulation of the levels of pro-inflammatory cytokines (IL-1β, IL-6 and TNF-α) and pro-apoptotic factor (P53) in the jejunum of piglets. In fact, maternal OH-SeMet supplementation in pigs during gestation was demonstrated to improve the offspring’s intestinal antioxidant capacity and reduce the inflammation level by suppressing NF-κB and ERK/Beclin-1 signalling [25]. It was suggested that maternal OH-SeMet supplementation during gestation might be beneficial for the immune function of their offspring by mitigating inflammation, autophagy, and ER stress levels in the thymus and spleen [26]. In particular, it was also shown that maternal (sows) OH-SeMet supplementation was able to alleviate ROS-induced intestinal ER stress by improving the expression of SELS and GPX4 in their offspring [27] as well as mitigating ROS-induced immunological stress by increasing the antioxidant capacity and altering the expression of inflammation-related genes and selenotranscriptome in immune organs [28]. OH-SeMet improved the antioxidant performance and immune function of gilts and changed the structure of the intestinal microbiota [29]. OH-SeMet was also reported to ameliorate chronic heat stress-induced porcine splenic damage via the activation of Nrf2/Keap1 signalling and the suppression of NF-κB and STAT signalling [30] and improved meat quality through the optimal skeletal metabolism and selenoprotein expression of pigs under chronic heat stress [31]. It seems likely that OH-SeMet, characterised by increased assimilation from the diet and providing an enhanced Se status in poultry [32,33] and pigs [34] in comparison to sodium selenite or SeY, could provide improved AO defences, improve gut health, and show anti-inflammatory properties under commercially relevant stress conditions. The organic Se story is quickly developing in poultry, pig, and ruminant nutrition [35] and will receive more attention in future to optimise the dietary supplementation of poultry and farm animals under commercially relevant stress conditions of egg and meat production.

Two papers in this Issue were devoted to polyphenolic sources in pig and poultry diets. It should be mentioned that polyphenolics and their role in the health maintenance of humans and animal/poultry production has received tremendous attention over the last 20 years. The effects of dietary *Eucommia ulmoides* leaf extract (ELE) supplementation on finishing pigs were characterised [36]. Specifically, 0.2% ELE supplementation did not affect growth performance but tended to reduce the backfat thickness of finishing pigs (*p* = 0.07). Interestingly, dietary ELE was shown to improve AO defences, as evidenced by increased SOD and GPX activities and decreased MDA in both serum and muscles and affected lipid metabolism in pigs [36]. The effects of dietary *Radix isatidis* (a ubiquitous botanical specimen in China) residual material (RIHR) on laying hens (including their serum biochemistry, egg quality, and intestinal health) have been studied [37]. The anti-inflammatory actions of RIHR in the ilea and ceca of laying hens were shown to be associated with improved egg quality (increased eggshell thickness, Haugh unit, and protein height). Furthermore, the total antioxidant capacity (T-AOC) of the yolk was also improved. The authors suggested that RIHR could directly affect the intestinal tract of laying hens to inhibit the expression of inflammatory factors (NF-κB, COX2, and IL-1β) and prevent inflammation [37]. Two review papers in this Issue addressed important questions related to the regulatory actions of polyphenolics in redox status maintenance in animals. The effects of dietary curcumin supplementation on the growth performance, serum antioxidant status, intestinal morphology, and meat quality of broiler chickens were evaluated through a meta-analyses of recent data [38]. Dietary curcumin supplementation was demonstrated to improve AO defences, as evidenced by significantly increased serum AO enzyme (SOD, CAT, and GPX) activities and decreased lipid peroxidation (MDA) in serum. Curcumin had protective effects on gut integrity and health, indicated by a significantly decreased crypt depth (CD), an increased villus height (VH), and an enhanced VH/CD ratio in the duodenum. Dietary curcumin also showed positive effects on meat quality, as indicated by increased carcass yield and colour (L*, a*, and b*) in meat and decreased cooking loss and fat and MDA content in the meat. Furthermore, dietary curcumin supplementation was able to enhance daily weight gain and improve the feed conversion ratio [38]. Similar conclusions have been made previously, indicating that curcumin can improve chicken growth, increase the egg production rate of laying hens, and, most importantly, partly mitigate the negative effects of heat stress on the production performance of poultry and livestock [39]. A recent (16 October 2025) literature search on PubMed (curcumin[Title]) AND (review[Title/Abstract]) gave 1408 hits, while adding “poultry” to the same search ((curcumin[Title]) AND (review[Title/Abstract])) AND (poultry[Title/Abstract]) gave only 5 results and (curcumin[Title]) AND (poultry[Title/Abstract] OR chicken[Title/Abstract]) gave 80 results. This indicates that curcumin studies in poultry are still in their infancy. Low solubility, difficulty in oral absorption, low biological utilisation, and the toxicity of curcumin compounds [40] restrict the widespread commercial applications of curcumin in poultry and animal production. It should be mentioned that the interest in the health-promoting properties of curcumin in humans is very high. Protective effects of curcumin have been shown in cognitive ageing [41], neurodegenerative diseases [42], including dementia [43], as well as in tuberculosis [44]. Curcumin was found to show anti-inflammatory actions by suppressing NF-κB signalling, attenuating mitochondrial ROS and ER stress, and disrupting inflammasome complex assembly [45]. However, its action is context-dependent and, in certain conditions, curcumin could promote pyroptosis by stabilising NLRP3 through the inhibition of Smurf2-mediated ubiquitination [45]. In a critical umbrella review of intervention meta-analyses related to the multiple health outcomes of curcumin in clinical practice, 25 recent studies were evaluated [46]. The findings showed that curcumin has positive effects on inflammatory markers and oxidative stress related to emotional and cognitive function, musculoskeletal diseases, ulcerative colitis, liver and kidney function, rheumatoid arthritis, and other diseases. However, the authors concluded that the overall methodological quality of the studies was relatively poor [46]. Furthermore, there are still some deficiencies in curcumin research that need to be strengthened in subsequent studies. Firstly, the drug loading capacity of the existing preparations is generally low and this leads to insufficient drug concentration, making it difficult to achieve the expected therapeutic effect [47]. Concurrently, the therapeutic potential of curcumin can be increased by exploring innovative targeted drug delivery strategies, improved formulation systems, and effective combination therapies. These modalities can promote its timely application in the treatment of various diseases and provide new avenues for therapeutic approaches [47].

In another paper published in this Issue, the anti-inflammatory properties of silymarin in various in vitro and in vivo model systems have been characterised [48]. The main anti-inflammatory mechanisms of silymarin (SM) and its main constituent silibinin/silybin (SB) are shown to be attributed to the inhibition of TLR4/NF-κB-mediated signalling pathways and the downregulated expression of pro-inflammatory mediators, including TNF-α, IL-1β, IL-6, IL-12, IL-23, CCL4, CXCL10, etc. Importantly, SM/SB was demonstrated to upregulate anti-inflammatory cytokines (IL-4, IL-10, IL-13, TGF-β, etc.) and lipid mediators involved in the resolution of inflammation. The major regulatory points of inflammation and resolution are show in Figure 1.

The inflammatory properties of SM/SB were proven in various in vitro model systems based on immune (macrophages and monocytes) and non-immune (epithelial, skin, bone, connective tissue, and cancer) cells. This was also confirmed in many different in vivo models, including toxicity models, non-alcoholic fatty liver disease, ischemia/reperfusion models, stress-induced injuries, ageing and exercising models, wound healing, and many other relevant model systems. It was concluded that the anti-inflammatory activities of SM/SB are key drivers of the health-promoting properties of these phytochemicals [48]. This was confirmed in other studies [49,50] with wide application in medical [51,52] and animal/poultry [53] sciences. In general, polyphenolics have received a great deal of attention over the last 20 years. However, their comparatively low availability and fast metabolic alterations in the body are major restrictions to their wide applications in medical and agricultural practices [49]. Of note, acute inflammation is an essential part of the immune system’s defence strategy, contributing to the creation of hostile conditions for pathogens and regulating the healing process. However, when inflammation resolution is dysregulated, chronic inflammation could lead to tissue damage and the development of various disease in humans and animals. In this context, disturbances in redox homeostasis due to oxidative stress and the dysregulation of the antioxidant defence network are important elements in over-inflammation in humans and farm animals/poultry. There are a great number of stress factors in human life and animal/poultry production (Table 1) leading to excessive inflammation, compromising health and promoting various diseases [1,48].

In conclusion, the papers published in this Issue further enhance the data supporting the important roles of dietary supplements, including vitamins, minerals, and polyphenolics, in redox balance maintenance to provide optimal antioxidant defences in commercially relevant stress conditions of meat and egg production.

Looking ahead, it is important to mention the increasing awareness related to the possible roles of redox balance and inflammatory responses in modern broilers and layers. The continued selection of broiler birds with a fast growth rate and increased breast muscle proportion has affected chicken physiology and biochemistry, making them more susceptible to various commercially relevant stresses [1]. It has been shown that there are distinctions in the skeletal muscle development between laying hens and broilers. Even under optimal feeding conditions, the weight of 6-week-old broiler chickens is more than five times that of egg-type chickens [54] and this significant difference leads to considerable physiological and genetic regulatory changes in broiler chickens. Fast growth is correlated with increased oxidative stress and reduced investment in homeostatic maintenance and repair [55]. Recently, it has been suggested that fast-growing broilers use different pathways than slow-growing birds to maintain their redox balance during growth [56]. As a result of the intense selection for a fast growth rate and increased breast muscle proportion, a range of metabolic diseases have developed in broilers, including various myopathies. Recently, it has been shown that broiler breast mitochondrial content has approximately halved in the last 45 years due to broiler selection for growth rate and increased meat yield and represents one of the lowest contents recorded for the muscle of any eukaryotic species [57]. This, together with compromised vascular system development, is involved in the development of muscle myopathies (wooden breast, spaghetti meat, and white stripping) in modern broilers [58,59]. Therefore, oxidative homeostasis and redox balance maintenance in fast-growing broilers deserve more attention in future research.

The greatest stress for commercial layers/breeders is during the peak of egg production. Indeed, the major compounds of egg yolk are synthesised in the liver, which often works to its maximal ability, and any form of stress can cause a decrease in egg production, which often does not recover after the stress is removed. In the ovaries of high-yielding laying hens, the ovulation process accelerates the accumulation of ROS, thereby causing oxidative stress in the tissues [60]. It has been shown that a high ovulation rate (HOR) imposes oxidative stress on layers. Compared with a low ovulation (LOR) group (caused by delayed photostimulation), HOR hens were characterised by the reduced activity of SOD and GPX in the plasma, liver, and ovary, while the levels of MDA in the same tissues were increased [61]. Furthermore, ROS levels, MDA concentrations, and 8-hydroxy-2′-deoxyguanosine (8-OHdG) concentrations in ovarian tissue and follicular granulosa cells were substantially higher in the HOR layers [62]. A recent transcriptome analysis of ovarian tissues identified genes controlling energy homeostasis and oxidative stress as potential drivers of heterosis for egg number and clutch size in crossbred laying hens. The authors showed that genes upregulated in the crossbred hens were associated with oocyte maturation and improved oocyte competence, while genes downregulated in the crossbred hens were shown to be related to oxidative stress promotion [63].

In laying hens, the late laying period is characterised by oxidative stress as evidenced by excessive ROS accumulation, compromised antioxidant defence systems, and increased inflammation [64,65,66]. Therefore, in aged laying hens, damage to the intestinal barrier, triggering inflammation, together with compromised AO defences, could result in severe OS, which further increases intestinal inflammation, disrupts intestinal integrity, and reduces nutrient absorption [64]. In addition, eggshell gland inflammation was shown to induce significant decreases in eggshell thickness and mechanical properties with structural deteriorations [67]. Furthermore, oxidative stress can increase inflammation in the eggshell gland. For example, LPS treatment was shown to increase the NF-κB pathway and inflammatory cytokine levels (TNF-α, IFN-γ, IL-1β, and IL-2) in eggshell glands [68]. It seems likely that eggshell gland, liver [69], and kidney [70] respond in a similar way to LPS-induced OS by increasing inflammation. Therefore, more attention should be paid to redox balance regulation via the affecting vitagenes [1,49] and transcription factors (e.g., Nrf2 [71] and NF-κB [72]), with special attention to hormesis [73] in ageing layers, in order to find an effective way to maintain redox homeostasis and decrease the decline in egg production and quality during the second part of the production period.

It seems likely that the application of vitagene-regulated compounds in water in the poultry [1,49] and pig [74,75,76] industries would be an important link between nutritionists and veterinarians, helping them to devise fast response systems to handle various stresses under the commercial conditions of egg and meat production [1].

## Figures and Tables

**Figure 1:**
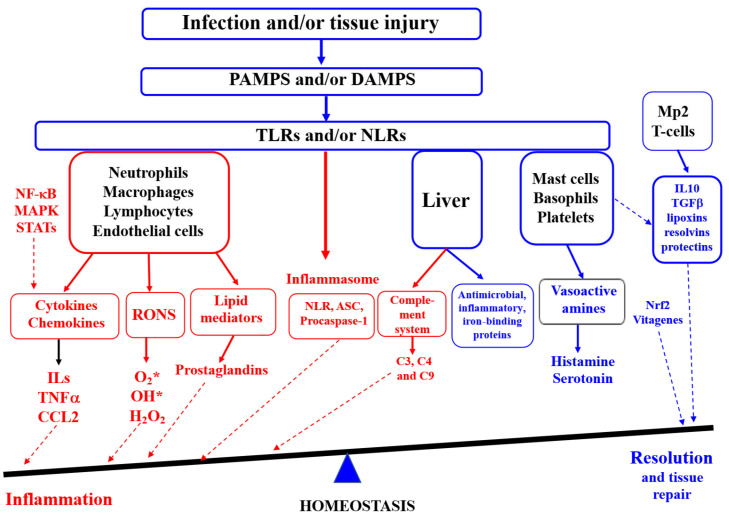
Inflammation and resolution (adapted from [48]). (ASC—adaptor molecule apoptosis-associated speck-like protein containing a CARD; CCL2—chemokine (C-C motif) ligand 2; DAMPS—damage-associated molecular patterns; IL-10—interleukin 10; MAPK—mitogen-activated protein kinases; STAT—signal transducer and activator of transcription; Mp2—type 2 macrophages; NLR—nucleotide oligomerization domain (NOD)-like receptors; PAMPS—pathogen-associated molecular patterns; TGF—transforming growth factor; RONS—reactive oxygen and nitrogen species; NLR—nucleotide-binding domain, leucine-rich repeat containing; TNFα—tumour necrosis factor alpha; TLRs—toll-like receptors).

**Table 1:** Inflammatory factors in humans and poultry (adapted from [48]).

Category	Pro-Inflammatory Factors	
	Humans	Poultry
Physical factors	Radiation, UV, hyperthermia, hypothermia, trauma	Hyperthermia, hypothermia, trauma, increased stocking density
Chemical factors	Asbestos, heavy metals, organic toxicants, dust, lipopolysaccharides	Heavy metals, mycotoxins, ammonia, CO, dust
Biological factors	Bacterial infection, viral infection, fungal infection	Bacterial infection, viral infection, fungal infection
Unhealthy lifestyle	Smoking, alcohol, high-calorie diet, stress, sedentary lifestyle	Restricted movement (cage housing), nutrient deficiency
Chronic diseases	Obesity, diabetes, hyperglycaemia	Chronic respiratory disease

## Data Availability

No new data were created or analyzed in this study. Data sharing is not applicable to this article.

## Abbreviations

The following abbreviations are used in this manuscript:

8-OHdG	8-hydroxy-2′-deoxyguanosine
25-OH-D3	25-Hydroxycholecalciferol
ADG	average daily gain
AO	antioxidant
CAT	catalase
CCL4	chemokine (C-C motif) ligand 4
CXCL10	C-X-C motif chemokine ligand 10
COX2	cyclooxygenase-2
ELE	Eucommia ulmoides leaf extract
ER	endoplasmic reticulum
ERKs	extracellular signal-regulated kinases
GSH	glutathione
GPX4	glutathione peroxidase 4
GPX6	glutathione peroxidase 6
GSH-Px	glutathione peroxidase
HOR	high ovulation rate
IFN-γ	interferon-gamma
IL	interleukin
ITMs	inorganic trace minerals
LOR	low ovulation rate
LPS	lipopolysaccharide
MDA	malondialdehyde
NF-κB	nuclear factor kappa-light-chain-enhancer of activated B cells
NLRP3	nucleotide-binding domain, leucine-rich-containing family, pyrin domain-containing-3
Nrf2	nuclear factor erythroid-2 related factor 2
OH-SeMet	hydroxy-selenomethionine
OTMs	organic trace minerals
OS	oxidative stress
RIHR	Radix isatidis residual material
ROS	reactive oxygen species
SELENON	selenoprotein N
SELS	selenoprotein S
SOD	superoxide dismutase
SB	silibinin/silybin
SM	silymarin
SeNa	sodium selenite
SeY	selenium-enriched yeast
STAT	signal transducers and activators of transcription
T-AOC	total antioxidant capacity
TGF-β	transforming growth factor-beta
TNF-α	tumour necrosis factor-alpha
TLR4	toll-like receptor 4
TXNRD	thioredoxin reductase
ZO-1	zonula occludens-1
